# Predictors of Risk Perception Among General Practitioners and Paediatricians Concerning Potential Health Effects of Exposure to Electromagnetic Fields

**DOI:** 10.1002/bem.70047

**Published:** 2026-02-26

**Authors:** Katharina Lüthy, Felix Forster, Claudia Riesmeyer, Lyn Ermel, Katja Radon, Tobias Weinmann

**Affiliations:** ^1^ Institute and Clinic for Occupational, Social and Environmental Medicine LMU University Hospital, LMU Munich Munich Germany; ^2^ Department of Media and Communication LMU Munich Munich Germany

**Keywords:** electromagnetic fields, general practitioners, Germany, paediatricians, risk assessment

## Abstract

Scientific evidence for health issues due to exposure to electromagnetic fields (EMF) is limited but there is considerable concern in the population about such effects. Physicians are seen as an important multiplier to the general population. The presented work intends to identify predictors of risk perception concerning EMF among general practitioners (GPs) and paediatricians. A cross‐sectional study was carried out in 2023 among 292 (response rate: 6%) GPs and paediatricians across Germany. Logistic regression modelling was applied to examine the relationship between different variables (technology acceptance, media health literacy, conspiracy belief, trust in organisations/institutions and environmental worry) and the physicians' health‐related risk perception regarding EMF. Ninety‐one participants (31%) indicated to believe in health issues as a consequence of EMF exposure. Higher EMF risk perception was indicated by physicians with high conspiracy belief compared to their peers with less conspiracy belief (odds ratio [OR]: 2.92; 95% confidence interval [CI]: 1.81–4.13). High trust in bodies like WHO (OR: 0.57; 95% CI: 0.35–0.82) or the Federal Office for Radiation Protection (OR: 0.50; 95% CI: 0.28–0.76) was associated with lower EMF risk perception. Overall, we observed considerable evidence that conspiracy belief and trust in organisations may predict EMF risk perception. Bioelectromagnetics. 00:00–00, 2026. © 2026 Bioelectromagnetics Society.

## Summary

- There are discrepancies between the level of scientific knowledge about electromagnetic fields (EMF) and EMF risk perception in the general public. For communicating scientific evidence to the public, it is important to understand physicians' risk perception and its determinants.
- Conspiracy belief and low trust in bodies like WHO predicted higher EMF risk perception among general practitioners and paediatricians.
- The study contributes to a deeper understanding of the risk perception of EMF among physicians and contributes to improving risk communication.

## 1 Introduction

In modern societies, exposure to electromagnetic fields (EMF) is ubiquitous and thus of very high relevance for public health (Götte and Ludewig 2019; Regrain et al. 2020). Usually, the arising exposure levels are well below legally prescribed limits though, while the scientific evidence for pronounced health issues due to EMF exposure below regulatory limits is scarce (Federal Office for Radiation Protection 2023a, 2023b; International Agency for Research on Cancer IARC 2002, 2013; Röösli et al. 2024). There are discussions about neurodegenerative diseases like Alzheimer or Parkinson's disease, childhood leukaemia, brain tumours and cancer in general (Federal Office for Radiation Protection 2023b; Frei et al. 2013; Huss, Spoerri, Egger, & Röösli 2009; International Agency for Research on Cancer IARC 2002, 2013; Karipidis et al. 2024), but so far there are no consistent findings (Federal Office for Radiation Protection 2023b; Karipidis et al. 2024; Scientific Committee on Health, Environmental and Emerging Risks (SCHEER) 2025). In addition to that, systematic reviews point out that available research suggests that there is no or only very low indication for an association between EMF exposure below guideline values and self‐reported non‐specific symptoms like tinnitus, migraine, headache, sleep disturbances, exhaustion, fatigue or nervousness (Bosch‐Capblanch et al. 2024; Durusoy et al. 2017; Röösli et al. 2024). Concerning other outcomes like cognitive impairment or effects on female reproductive/male fertility, there is also only very little evidence for an association with EMF exposure below legally prescribed limits (Benke et al. 2024; Johnson et al. 2024; PW Kenny et al. 2024; SCHEER 2025).

However, there is considerable concern in the general population about health effects of EMF (Regrain et al. 2020). In Germany for instance, a routine survey of the Federal Office for Radiation Protection implies that about one fifth of the general population is concerned about potential effects of EMF exposure on their health (Götte and Ludewig 2019). The latest version of the survey—performed in 2022—reported that between 18% and 23% of the population have health concerns about radiation occurring from smartphones, cell phone towers or high voltage lines (Federal Office for Radiation Protection 2022). Aside from that, the “Eurobarometer‐Study” from 2010 reported that 46% of the EU population is very concerned about possible health risks from EMF. In addition, only around 40% said that they were dissatisfied with the information given about this topic (TNS Opinion and Social 2010).

As the central pillar of primary care, general practitioners (GPs) and paediatricians are often the first point of contact in the health system for the general population. One of their roles is to transfer and explain scientific evidence to the public. This can be a difficult task since scientific publications are often very difficult to understand for laypersons. As a consequence, discrepancies between scientific statements and concerns may occur. Therefore, the communication of scientific knowledge in relation to EMF between GPs/paediatricians and their patients—or in the case of children, their parents—is of fundamental importance to avoid uncertainty within the general population (Huss and Röösli 2006). For example, a study that investigated where the general population would inquire about or search for information on possible health risks of mobile phones showed that GPs were mentioned second (after searching for information on the Internet) (Hartmann and Belz 2010). This professional group is therefore an important multiplier of health‐related specialist information (Blümel et al. 2024).

In previous studies carried out in Germany in 2024 and 2009 and in similar studies in Austria, the Netherlands and France rather little knowledge about potential EMF risks among physicians was observed. Moreover, GPs often indicated great concern and felt insufficiently informed on the subject of health and EMF (Berg‐Beckhoff et al. 2009; Forster et al. 2024; Forster et al. 2025; Kowall et al. 2010; Lambrozo et al. 2013; Leitgeb et al. 2005; Slottje et al. 2017).

The German study from 2009 indicated that additional education in alternative medicine is so far the only known predictor for EMF worries (Berg‐Beckhoff et al. 2009; Kowall et al. 2015). Apart from that, little to nothing is known about predictors of risk perception concerning potential health effects of exposure to electromagnetic fields among GPs and paediatricians.

If radiation protection authorities or federal offices want to address worries, close gaps in knowledge and eliminate incorrect views regarding potential health effects of EMF, both among the general public and among GPs and paediatricians, it is necessary to understand individuals' state of knowledge and risk perception and the factors that determine them. Such knowledge may help to improve communication of scientific knowledge on EMF exposure and health by a better understanding of predictors of EMF‐related concern and facilitating the development of specific communication formats and materials that is tailored to specific groups.

For this reason, we conducted a survey among a representative sample of German GPs and paediatricians. The objective of the survey was to identify predictors of risk perception concerning EMF in GPs and paediatricians.

Specifically, we aimed to assess the following hypotheses:

A higher level of technology acceptance is associated with lower EMF risk perception.

A higher level of media health literacy and competence in digital media and technologies is associated with lower EMF risk perception.

A higher level of conspiracy belief is associated with higher EMF risk perception.

Trust in organisations/initiatives/institutions is associated with EMF risk perception (direction of association depends on type of organisation/initiative/institution).

EMF risk perception is associated with general environmental worry.

## 2 Materials and Methods

### 2.1 Analysed Variables

We performed a cross‐sectional study that collected data in the period from February up to April 2023. A stratified random sample—by federal state and additional education in homeopathy—of 3000 GPs and 2000 paediatricians in Germany was drawn from the German Federal Medical Registry (Hereafter: Sample). Physicians participating in statutory health insurance are mandatory members of this registry. All 5000 GPs and paediatricians were invited to fill in an online questionnaire.

#### 2.1.1 Outcome

Physicians' risk perception regarding potential health impacts caused by EMF was the main outcome of interest. It was measured as the respondents' belief that “there are people who develop health issues as a consequence of EMF exposure within legally prescribed limits”. This explicit question was chosen to ensure comparability to the previous study from 2009 and is based on relevant comparable literature (Berg‐Beckhoff et al. 2009). A 5‐point‐Likert‐Scale was used and the variable was dichotomised, defining answers above the central option as high risk perception and the central answer option or lower as low risk perception.

#### 2.1.2 Predictors

There were several predictors of interest, namely general environmental worry, technology acceptance, media health literacy, conspiracy belief, and the trust in various organisations/institutions.

Technology acceptance was measured by asking about physicians' agreement with 12 statements regarding 5 G, for example, “Compared to 3 G and 4 G, using 5 G services will save time.” (Akbari et al. 2020). A 5‐point‐Likert‐Scale was applied and a sum score was calculated from the 12 items (sum scores of the individual Likert scale values of the respective items: minimum value: 0, maximum value: 48).

Media health literacy was measured based on a questionnaire (Fleary 2022) in which three health‐related images were shown to the participants and questions regarding their content were asked. Below the images were a total of 12 questions, each with one correct answer. Each correctly answered question was awarded one point. For example, participants received one point if, in the case of an image showing a child next to a smoking adult, they checked the box indicating that the intention of the image was to encourage someone to quit smoking. Of all 12 questions a sum score was formed (sum value of the individual correct answers of the respective items: minimum value: 0, maximum value: 12).

Conspiracy belief was measured by asking about physicians' agreement with five statements, for example, “The truth is that the association between health issues and EMF is hidden from the public” (Jolley and Paterson 2020). A 5‐point‐Likert‐Scale was used and a sum score was calculated (sum scores of the individual Likert scale values of the respective items: minimum value: 0, maximum value: 20).

Trust in organisations (World Health Organization [WHO], Federal Office for Radiation Protection, International Commission on Non‐Ionizing Radiation Protection [ICNIRP], the Federation of German Consumer Organisations, the German Medical Association/State Chambers of Physicians) and institutions (Federal government, politicians, political parties, courts of law, large companies, police, armed forces, media, science, most other people) was measured with 5‐point‐Likert ‐Scales (mean values using the 5‐point Likert scales: minimum value: 0, maximum value: 4). The operationalisation of trust in institutions is based on a distinction established in communication studies between media, social, political, and societal trust (Steindl et al. 2023). The term “media” encompasses trust in all different media objects, such as media brands, media types or mainstream journalistic media, without differentiating between different offerings. “Science” encompasses the perception of all individual and collective scientific actors. Finally, “most other people” encompasses the perception of public opinion and trust in public opinion at the societal level. These institution actors disseminate messages in a wide variety of forms at different levels (individually, as members of organisations) and thus address different audiences.

In addition to that, trust in “EMF‐critical” institutions (pleas from EMF sceptical physician initiatives, citizen initiatives against the construction of mobile phone base stations, Institute for Applied Ecology) was measured—with 5‐point‐Likert ‐Scales (mean values using the 5‐point Likert scales: minimum value: 0, maximum value: 4)—too.

Higher values indicated higher technology acceptance, higher media health literacy, higher tendency towards conspiracy belief, as well as higher trust in organisations/institutions/initiatives.

General environmental worry was measured by the question: “Do you worry about your personal health because of…” regarding the following six items: air pollution, traffic noise, drinking water pollution, impacts of climate change, resistance to antibiotics, and pesticides. A 5‐point‐Likert‐Scale was used and physicians who answered for at least three items above the central answer option were considered as having high environmental worry, while worry was considered low otherwise (dichotomous value: high environmental worries vs not worried).

#### 2.1.3 Potential Confounders

Sex (male, female), age group (≤ 40 years, 41–50, 51–60, > 60), type of physician (GP, paediatrician), state (16 German states) as well as the size of the town in which the physician's place of work is located (large town (≥ 100,000 inhabitants), medium town (< 100,000 and ≥ 20,000), small town (< 20,000 and ≥ 10,000), village (< 10,000 and ≥ 5000), rural municipality (< 5000), and additional education in alternative medicine were included as potential confounding factors. Data on those factors came from the Federal Medical Registry. In addition, education in alternative medicine was included in the questionnaire. Registry and questionnaire data were combined by assuming that additional education is available if it is reported by at least one source, that is, registry or questionnaire.

In previous studies on EMF risk perception from the perspective of GPs, the presence of additional training in alternative medicine consistently emerged as a relevant factor (Berg‐Beckhoff et al. 2009; Huss and Röösli 2006; Leitgeb et al. 2005; Slottje et al. 2017). At the same time, a precious study in Germany showed that the associations can differ depending on the type of additional training (Kowall et al. 2015). Three predefined types of alternative medicine were available in the German Federal Medical Registry and therefore included in the study: homeopathy (HP), acupuncture (AC), and naturopathic treatment (NPT). Due to small number of participants with an additional education in AC and NPT, it was assumed that considering all three types of alternative medicine individually would be inappropriate. Therefore, they were combined to three different versions: HP (yes, no), HP or AC (yes, no), HP or AC or NPT (yes, no). If another relevant additional education in alternative medicine was mentioned in the questionnaire, HP or AC or NPT was defined as available, while the other two variables were not changed.

### 2.2 Statistical Analysis

Descriptive statistics are reported for outcome, predictors, and potential confounders.

Logistic regression was conducted to estimate the relationship between the various potential predictors and the outcome (EMF risk perception). Odds ratios (OR) with corresponding 95% confidence intervals (CI) were obtained from the models. For every predictor, a separate model was estimated. Sum scores were standardised ((sum score – mean value)/standard deviation) before inclusion in regression models. General environmental worry was included as a dichotomous variable, while the original Likert scale values (values 0–4) were used for the variables describing the trust in organisations, institutions and initiatives. All models were adjusted for sex, age group, type of physician, state, size of town, and additional education in alternative medicine. Since there were three definitions of additional education in alternative medicine, all models were calculated three times, once for each definition. Due to the extremely low number of missing data, we performed complete case analyses.

Data management and analysis was performed using R version 4.1.1 (R Core Team 2021). Regression models were calculated using package “rstanarm” (Goodrich and Gabry 2020), which uses the Bayesian analysis software Stan (Carpenter et al. 2017). For each model, four chains of 2000 samples each (1000 of them being warm‐up samples) were drawn, leading to 4000 samples available for estimation. Weakly informative default priors from the rstanarm package were used. Diagnostic criteria (effective sample size, R_hat, tree depth, energy, trace plots) were checked and indicated no problems with the sampling procedure (Betancourt 2017). Based on Lash et al. (Lash et al. 2009) and the package ‘episensr’ (Haine and Wood 2023) a simple investigation of a potential selection bias was carried out. It is based on the 2 × 2‐table and four selection probabilities—one for each cell of the table. These selection probabilities describe what percentage of the sample (within each cell) participated in the study. We made assumptions regarding pairwise comparisons of selection probabilities, that is, for every pair of selection probabilities, which of them is higher or lower. Based on the pairwise comparisons, we used the ‘episensr’ package to estimate the direction of bias.

## 3 Results

In total, 292 physicians (response rate: 6%) answered the online questionnaire—henceforth referred as “study population”. Of these, 141 (48%) were GPs and 151 (52%) were paediatricians.

Just over half of the participating physicians were female (51%). The majority of the physicians were between 41 and 60 years old. In comparison to the sample (all 5000 invited physicians), where over 60% of the persons were over 50 years old, the study population was somewhat younger, with just over 50% being under 50 years old.

69% of participants' practices were located in large or medium‐sized towns. There were hardly any differences compared to the entire sample in this respect.

In total, 25% of the study population had an additional education in HP, AC, or NPT.

In our study population, a slightly higher proportion of physicians with additional education was observed compared to the sample. Overall, however, it can be said that the sociodemographic distribution of our study population was broadly comparable to our sample, in particular the distribution of gender and site of physician's practice (Table 1).

**Table 1:** Description of sociodemographic variables in total and in relation to the main outcome. Sample: all invited physicians. Study population: Physicians who answered the questionnaire.

	Sample	Study population		
	Total *N*: 5000 *n* (%)	Total *N*: 292 *n* (%)	Belief in health issues as EMF consequence *N* *: 291 *n* (%)	
			Yes 91 (31.3)	No 200 (68.7)
Age				
≤ 40	525 (10.5)	44 (15.1)	11 (12.1)	33 (16.5)
41–50	1304 (26.1)	113 (39.7)	36 (39.6)	77 (38.5)
51–60	1767 (35.3)	80 (27.4)	27 (29.6)	52 (26.0)
≥ 60	1404 (28.1)	55 (18.8)	17 (18.7)	38 (19.0)
Sex				
Female	2730 (55.6)	149 (51.0)	49 (53.9)	99 (49.5)
Site of physician's practice				
Large town	1796 (35.9)	104 (35.6)	23 (25.3)	80 (40.0)
Medium town	1668 (33.4)	98 (33.6)	33 (36.3)	65 (32.5)
Small town	750 (15.0)	39 (13.4)	12 (13.2)	27 (13.5)
Village	540 (10.8)	29 (9.9)	14 (15.4)	15 (7.5)
Rural municipality	246 (4.9)	22 (7.5)	9 (9.9)	13 (6.5)
Additional education				
HP	134 (2.7)	21 (7.2)	16 (17.6)	5 (2.5)
HP or AC	326 (6.5)	54 (18.5)	32 (34.1)	22 (11.0)
HP or AC or NPT	536 (10.7)	74 (25.3)	42 (46.2)	32 (16.0)

Abbreviations: AC, acupuncture; EMF, electromagnetic fields; HP, homoeopathy; NPT, naturopathic treatment.

* 1 (0.3%) missing value in variable “Belief in health issues as EMF‐consequence.”

Regarding the outcome, 200 (69%) physicians indicated that they do not believe in health issues as a consequence of EMF exposure. This proportion did not meaningfully differ between GPs (68.8%) and paediatricians (68.9%). There were no differences in age distribution between physicians who believed in health issues as EMF consequences and those who did not. In addition to that, there was also no difference concerning sex. Looking at the location of the medical practice, it became apparent that a larger proportion of physicians who believe in EMF health consequences tended to work in smaller communities. However, a major difference can be seen with regard to additional education in alternative medicine: While nearly half of the physicians who believed in health issues as EMF consequence have received further education in alternative medicine (46.2%), only 16.0% of the physicians who did not believe in EMF health effects have additional education in HP or AC or NPT.

The average sum score for technology acceptance was 21.8 out of 48 (on average, physicians picked the central answer option or the one below) and 9.8 out of 12 for media health literacy (Table 2).

**Table 2:** Description of potential predictors.

	Missings *n* (%)	Total mean (SD)	GPs mean (SD)	Paediatricians mean (SD)
Technology acceptance	41 (14.0)	21.8 (10.8)	22.5 (10.4)	21.2 (11.2)
Media health literacy	39 (13.4)	9.8 (1.5)	9.8 (1.4)	9.8 (1.6)
Conspiracy belief	42 (14.4)	4.2 (4.4)	4.0 (4.3)	4.3 (4.6)
Trust in organisations				
WHO	41 (14.0)	3.4 (0.8)	3.2 (0.9)	3.4 (0.7)
Federal Office for Radiation Protection	41 (14.0)	3.5 (0.7)	3.6 (0.6)	3.5 (0.7)
ICNIRP	47 (16.1)	3.0 (0.9)	2.9 (1.0)	3.0 (0.9)
Federation of German Consumer Organisations	41 (14.0)	3.1 (0.8)	3.1 (0.8)	3.1 (0.8)
German Medical Association	41 (14.0)	3.1 (0.9)	3.1 (0.9)	3.1 (0.9)
Trust in initiatives				
Institute for Applied Ecology	50 (17.1)	2.1 (1.0)	2.2 (1.1)	2.1 (1.0)
Pleas from EMF sceptical physician initiatives	49 (16.8)	2.0 (1.0)	1.9 (1.1)	2.2 (1.0)
Citizens' initiatives	43 (14.7)	1.1 (1.0)	1.1 (1.0)	1.0 (0.9)
Trust in institutions				
Federal government	43 (14.7)	2.5 (0.9)	2.4 (1.0)	2.6 (0.9)
Politicians	43 (14.7)	1.8 (0.8)	1.8 (0.9)	1.9 (0.8)
Political parties	43 (14.7)	1.8 (0.8)	1.8 (0.9)	1.8 (0.7)
Courts	43 (14.7)	3.0 (0.8)	3.0 (0.9)	3.1 (0.7)
Large companies	43 (14.7)	1.1 (0.8)	1.1 (0.8)	1.0 (0.8)
Police	43 (14.7)	3.1 (0.7)	3.0 (0.8)	3.1 (0.7)
Armed forces	43 (14.7)	2.7 (0.8)	2.7 (1.0)	2.7 (0.7)
Media	43 (14.7)	1.7 (0.9)	1.7 (0.9)	1.8 (0.9)
Science	43 (14.7)	3.4 (0.7)	3.4 (0.7)	3.4 (0.7)
Most other people	43 (14.7)	2.3 (0.9)	2.3 (0.9)	2.4 (0.8)
	*n* (%)	*n* (%)	*n* (%)	*n* (%)
Environmental worry: high	40 (13.7)	184 (73.0)	79 (66.9%)	105 (78.4%)

Abbreviations: EMF, electromagnetic fields; ICNIRP, International Commission on Non‐Ionizing Radiation Protection; SD, standard deviation; WHO, World Health Organisation.

Most of the participating physicians (73%) had a high environmental worry while the conspiracy belief was at a relatively low level with a mean value of 4.2 out of a possible maximum of 20. With mean values of 3.4 (SD: 0.9) and 3.5 (SD: 0.7) out of a maximum of 4, WHO and Federal Office for Radiation Protection were the organisations the participants trusted the most. Trust in citizens' initiatives against the construction of mobile phone base stations, on the other hand, was very low (mean: 1.1; SD: 1.0). Except for environmental worry—where the proportion of worried paediatricians (78.4%) was higher compared to GPs with a proportion of 66.9%—the results of the possible predictors for both professional groups hardly differed. Environmental factors the physicians worried most were antibiotic resistance with a share of 79.8% indicating worries or high worries, effects of climate change (proportion of concern or strong concern: 78.6%) and pesticides (proportion of concern or strong concern: 67.8%). In contrast to that, the environmental topics air pollution, water pollution and traffic noise seemed a bit less relevant with corresponding concern proportions of 59.6%, 41.3% and 32.1%.

With an OR of 0.81 (95% CI: 0.57–1.09), physicians with higher technology acceptance tended towards lower EMF risk perception than those with lower technology acceptance (Table 3). The same (with a stronger level of evidence) applies to physicians who reported higher trust in organisations like WHO or Federal Office for Radiation Protection—the higher the trust in these organisations, the lower the EMF risk perception (WHO: OR: 0.57; 95% CI: 0.35–0.82; Federal Office for Radiation Protection: OR: 0.50; 95% CI: 0.28–0.76).

**Table 3:** Adjusted logistic regression model for the association of exposures and EMF risk perception.

	Belief in health issues as EMF‐consequence OR (95% CI)*
Technology acceptance	0.81 (0.57–1.09)
Media health literacy	1.09 (0.78–1.47)
Conspiracy belief	2.92 (1.81–4.13)
Trust in organisations	
WHO	0.57 (0.35–0.82)
Federal Office for Radiation Protection	0.50 (0.28–0.76)
ICNIRP	0.85 (0.58–1.16)
Federation of German Consumer Organisations	0.72 (0.47–1.05)
German Medical Association	0.84 (0.55–1.15)
Trust in initiatives	
Institute for Applied Ecology	1.52 (1.04–2.05)
Pleas from EMF sceptical physician initiatives	1.50 (1.04–2.03)
Citizens’ initiatives	2.82 (1.79–4.04)
Trust in institutions	
Federal government	0.95 (0.63–1.33)
Politicians	1.12 (0.72–1.64)
Political parties	1.12 (0.70–1.60)
Courts	0.67 (0.41–0.97)
Large companies	1.33 (0.82–1.97)
Police	0.94 (0.55–1.42)
Armed forces	1.12 (0.72–1.65)
Media	0.71 (0.48–0.99)
Science	0.76 (0.43–1.22)
Most other people	1.13 (0.75–1.59)
Environmental worry	1.08 (0.47–2.06)

Abbreviations: CI, confidence interval; EMF, electromagnetic fields; ICNIRP, International Commission on Non‐Ionizing Radiation Protection; OR, odds ratio; WHO, World Health Organisation.

* Adjusted for type of physician, age, sex, site of physician's practice, federal state and additional education defined as homoeopathy (yes/no).

In contrast, conspiracy belief showed one of the strongest associations with EMF risk perception. Higher risk perceptions were found in the population with higher conspiracy belief compared to their peers with lower tendency towards conspiracy belief (OR: 2.92; 95% CI: 1.81–4.13). Higher trust in all three mentioned “EMF‐critical” initiatives was associated with higher EMF risk perception (Institute for Applied Ecology: OR: 1.52; 95% CI: 1.04–2.05; Pleas from EMF sceptical physician initiatives: OR: 1.50; 95% CI: 1.04–2.03; citizens' initiatives: OR: 2.82; 95% CI: 1.79–4.04).

Besides, we observed no difference in EMF risk perception between physicians with higher general environmental worry compared to those with lower general environmental worry (OR: 1.08, 95% CI: 0.47–2.06).

Based on our assumptions with regard to the selection probabilities of the outcome and the predictors we considered most relevant based on the previous calculated logistic regression model (conspiracy belief and trust in WHO), we analysed the effects of a possible selection bias. This analysis of selection bias suggested different directions of bias (Table 4).

**Table 4:** Effects of a possible selection bias.

Assumptions regarding the selection probabilities	Bias
Conspiracy belief	
High EMF risk‐perception leads to higher study participation & high conspiracy belief leads to lower study participation	Towards 1
High EMF risk‐perception leads to lower study participation & high conspiracy belief leads to lower study participation	Away from 1
Trust in organisation: WHO	
High EMF risk‐perception leads to higher study participation & low trust in the WHO leads to lower study participation	Towards 1
High EMF risk‐perception leads to lower study participation & low trust in the WHO loads to lower study participation	Away from 1

Abbreviations: EMF, electromagnetic fields; WHO, World Health Organisation.

## 4 Discussion

The present study investigated predictors of EMF‐related risk perception in GPs and paediatricians. Our analysis confirmed the hypothesis that a higher level of conspiracy theories is associated with higher EMF risk perception. Furthermore, we observed some evidence for an association between the trust in certain organisations, initiatives or institutions, for example WHO or the Federal Office for Radiation Protection, and EMF risk perception. It could not be definitively confirmed whether higher technology acceptance and greater media literacy in healthcare are associated with a lower perception of EMF risk. Furthermore, no association was observed between general environmental worry and the perception of EMF risk.

Our study indicated that conspiracy belief and trust in various organisations/institutions—for example citizen initiatives against the construction of mobile phone base stations—are predictors of high EMF risk perception. High trust in the WHO or radiation protection authorities like the German Federal Office for Radiation Protection, on the other hand, were associated with lower EMF risk perception.

There are numerous conspiracy theories circulating in the media and social media that postulate a connection between EMF exposure and harmful health effects (Elzanaty et al. 2021). Some of them for example claim that 5 G weakens the immune system and EMF can lead to a deadly inflammation (Elzanaty et al. 2021). Especially in the time of the COVID‐19 pandemic, a massive amount of misinformation in this context led to uncertainties and anger, which in turn led to protests and acts of vandalism (Meese et al. 2020). Our results suggest that conspiracy belief could indeed be linked to higher EMF risk perception while trust in health authorities like WHO may be inversely associated with higher risk perception on EMF. Other studies suggest that initiatives aiming to increase community engagement and trust towards authorities by satisfying the needs for autonomy, competence and relatedness may reduce the prevalence of general conspiracy belief (Leonard and Philippe 2021). In addition, health authorities are recommended to be careful not to spread contradictory information, to be transparent in their decision‐making processes (Carlsen and Glenton 2016) and to invest in the implementation of community‐feedback‐mechanisms (Baggio et al. 2019; Leonard and Philippe 2021). Researchers and other experts in the field of conspiracy theories could also be encouraged to increase their media presence in order to disseminate reliable, scientifically sound information and thus counter conspiracy theories (Burstein 2003). Besides, trust in authority figures or organisations can be undermined by misinformation, uncovered past conspiracies, and the unequal treatment of minorities (Leonard and Philippe 2021). Therefore, to strengthen public trust in authorities and political decision‐makers, the principles of accountability and transparency should be supported and those responsible for misconduct may be held publicly accountable (Leonard and Philippe 2021).

Overall, however, regarding EMF it must be said, that further research is needed to investigate how a reduction in belief in conspiracy theories and higher trust in risk communication organisations (e.g., WHO or Federal Office for Radiation Protection) could be explicitly achieved in real life.

Risk perception among physicians with high environmental worry was as high as risk perception among physicians with low general environmental worry. Our results suggest that concern about effects of EMF on health are indeed a distinct phenomenon, and therefore should be considered separately and independent of environmental concern.

### 4.1 Compatibility with Other Studies

In the only other study among German GPs, the risk perception was determined by the question whether the participants believed that there are people who develop health issues as a consequence of EMF exposure (Berg‐Beckhoff et al. 2009). With 54% of agreement, this study reported higher values of EMF concern among GPs compared to our survey where 31% agreed. In contrast to our study, in the earlier study there was no explicit mention of legally prescribed limits and with about 43%, the proportion of physicians with additional education in alternative medicine in the earlier study was clearly higher than in our study. Furthermore, there is a difference in gender distribution between this study population and ours. Women were less frequently represented in the 2009 study (approximately 35%) than in ours. Participants in the previous study—who were all GPs—also indicated high levels of trust in WHO and Federal Office for Radiation Protection. The odds of high risk perception of physicians with high trust in WHO was 40% lower compared to those with low trust (Berg‐Beckhoff et al. 2009). While our findings concerning trust in organisations is thus in line with the previous study among German GPs, Berg‐Beckhoff and colleagues did not measure conspiracy belief. In fact, our study is the very first to identify conspiracy belief as a major determinant of high EMF risk perception. Furthermore, due to the different methods of participant recruitment, the different categorisation of the sociodemographic variables “age” and “location of medical practice” and the aspects just mentioned, a comparison between the two studies is generally limited.

Aside from that, this previous study was performed over 15 years ago. It is possible that EMF risk perception itself has decreased over time because maybe there is currently greater concern about other issues or maybe the perceived benefits of technologies that produce EMF outweigh the risks. A nationwide survey in Germany entitled “What role do magnetic fields play in the public perception of electricity grid expansion?” showed that the issues that worried study participants most about their health in 2022 were antibiotics/pesticides in food, air pollution and multi‐resistant bacteria (Götte and Ludewig 2024). In addition to that, in our study over 65% of the physicians had concerns or very strong concerns regarding pesticides, the effects of climate change and antibiotic resistance. The issue of EMF concerns seems to be fading into the background. Furthermore, the results from the 2022 study suggest that respondents, for example, rate the benefits of high‐voltage power lines as rather high or very high, with a total of 51.8% (for themselves personally) and 76.7% (for society) (Götte and Ludewig 2024). A qualitative study reported that mobile phone use is viewed more positively than mobile phone base station use, as mobile phones are used, for example, to stay in touch with family and friends (Link et al. 2024). It was also discussed whether the more positive view of mobile phones could be due to their much greater presence in our daily lives than mobile phone base stations (Link et al. 2024). These studies, for example, imply that the benefits of EMF‐generating technologies can outweigh the disadvantages.

Other studies indicate that the level of concern regarding various issues can change over time. A study from Belgium that was published in 2025 observed that already within 1 year concerns about pesticide sprays and mobile phone antennas were replaced by climate change and antibiotics in food among the five most frequently cited sources of concern (Ledent et al. 2025).

A study performed 2003 in Austria among GPs concluded that 33% of the participants answered the question “Do you think ‘electromagnetic pollution’ can cause illness?” with “yes” and overwhelming 95% agreed to some degree or even completely (Leitgeb et al. 2005). Furthermore, 96% of the GPs in this study agreed to some degree or even completely to the question “Do you think, ‘electromagnetic pollution’ together with other environmental factors can cause health problems?” (Leitgeb et al. 2005). It was not mentioned which environmental factors were meant here. Of particular note is the highly negative choice of the word ‘pollution’. Furthermore, each of the six possible answer options that was not explicitly ‘no’ was interpreted as agreement. A direct comparison of this study with ours is thus difficult.

A study conducted in France also reported that doctors considered some environmental issues more relevant than EMF‐related issues. For example, being near smokers, drinking from lead pipes, or living near a nuclear power plant was perceived as more worrying than living near high‐voltage power lines/cell phone base stations or regularly using a mobile phone (Lambrozo et al. 2013). However, a direct comparison to our outcome and our predictors is not possible here due to different questions and outcomes.

Another study from the Netherlands, that was published in 2017 reported that about 37% of the participating GPs agreed or rather agreed with the statement “Exposure to EMF can lead to health complains” (Slottje et al. 2017). This result hardly differs from our findings, but it has to be mentioned, that the wording of the question—especially the mentioning of “Electromagnetic hypersensitivity”—and the answer scale differ. Additionally, the explicit mentioning of legally prescribed limits was missing here, too.

Altogether, a detailed comparison between our study and other studies is very difficult. Here too, it becomes clear that studies are lacking and research gaps exist, specifically relating to the topic of EMF risk perception and its possible predictors.

### 4.2 Strengths and Limitations

It seems reasonable to assume that physicians who are concerned about EMF were more likely to participate in the study out of self‐interest, especially since “assessment of the risk of electromagnetic fields” was explicitly mentioned in the study invitation. On the other hand, concern about EMF could have led to a lower study participation because of avoiding electronic technologies like computers—participation in an online study could therefore be less likely. Hence, the outcome could have had influence on the probability of participation and—for example, in the case of increased participation among EMF‐concerned physicians—could have led to an overrepresentation of EMF concern.

In addition, it appears possible that physicians with high conspiracy belief or low trust in institutions like WHO may have been less likely to take part in this study as a result of low levels of credibility or meaningfulness of the study itself. Accordingly, influence of the two predictors conspiracy belief and trust in the WHO on study participation cannot be ruled out either.

As both exposures and outcome could have influenced study participation, selection bias cannot be ruled out. Depending on the assumptions, the ORs may be biased in different directions. However, the strength of the direction is difficult to assess, because we used dichotomised versions of our predictors for the bias investigation and confounders were not taken into account.

Looking at the socio‐demographic variables and potential confounders, their distributions were largely comparable in our total sample and the study population. There were however differences in the distribution of age and the existence of an additional education in alternative medicine. But in general, despite the low response rate, our study population socio‐demographically seems to be quite representative for the sample regarding many variables.

We are also aware that our study may have some limitations because of unconsidered confounders. To the best of our knowledge, we included all relevant confounders—based on similar relevant literature—and adjusted for them. Nevertheless, it cannot be ruled out that there are some other confounders that were not taken into account, especially since little is known about predictors in relation to EMF worries.

For recruitment, we were able to access extensive data from the Federal Medical Register. This enabled us to draw our sample from a source population that includes all physicians with only few exceptions. This suggests a high level of generalisability which, however, only applies to Germany.

### 4.3 Implications

To address EMF worries in physicians, it seems important to reduce conspiracy beliefs and increase the trust in institutions of risk assessment and management. In the long term this would also benefit public health via improved knowledge transfer and communication towards the general population. In this context, it would be interesting to investigate the relation between conspiracy belief and EMF risk perception among the general population.

## 5 Conclusions

We identified individuals' inclination towards conspiracy belief as a predictor of risk perception concerning EMF in physicians, even after adjustment for additional education in alternative medicine. A second important predictor was their trust in institutions like WHO or radiation protection authorities. These results, however, might have been influenced by selection bias, the direction of which depends on the specific assumptions on how these variables affected participation in the study.

## Ethics Statement

This study was performed in line with the principles of the Declaration of Helsinki. Approval was granted by the Ethics Committee at the Medical Faculty of LMU Munich, Munich, Germany (Project number: 24‐0204). The study was approved by the Ethics Committee at the Medical Faculty of LMU Munich. All participants gave informed consent, including for linking questionnaire and registry data.

## Consent

Informed consent was obtained from all individual participants which included linking questionnaire and registry data.

## Conflicts of Interest

The authors declare no conflicts of interest.

## Data Availability

The data is not available from the authors because the use of the registry data must be approved by the Federal Ministry of Health.
